# Expression profile of CREB knockdown in myeloid leukemia cells

**DOI:** 10.1186/1471-2407-8-264

**Published:** 2008-09-18

**Authors:** Matteo Pellegrini, Jerry C Cheng, Jon Voutila, Dejah Judelson, Julie Taylor, Stanley F Nelson, Kathleen M Sakamoto

**Affiliations:** 1 Department of Molecular, Cellular, and Developmental Biology, University of California, Los Angeles, USA; 2 Division of Hematology-Oncology, Department of Pediatrics, Kaiser Permanente Medical Center, Los Angeles, USA; 3 Department of Human Genetics, David Geffen School of Medicine at UCLA, Los Angeles, USA; 4 Division of Hematology-Oncology, Department of Pediatrics, Gwynne Hazen Cherry Laboratories, Department of Pathology and Laboratory Medicine, David Geffen School of Medicine at UCLA, Los Angeles, USA; 5 Division of Biology, California Institute of Technology, Pasadena, USA

## Abstract

**Background:**

The cAMP Response Element Binding Protein, CREB, is a transcription factor that regulates cell proliferation, differentiation, and survival in several model systems, including neuronal and hematopoietic cells. We demonstrated that CREB is overexpressed in acute myeloid and leukemia cells compared to normal hematopoietic stem cells. CREB knockdown inhibits leukemic cell proliferation *in vitro *and *in vivo*, but does not affect long-term hematopoietic reconstitution.

**Methods:**

To understand downstream pathways regulating CREB, we performed expression profiling with RNA from the K562 myeloid leukemia cell line transduced with CREB shRNA.

**Results:**

By combining our expression data from CREB knockdown cells with prior ChIP data on CREB binding we were able to identify a list of putative CREB regulated genes. We performed extensive analyses on the top genes in this list as high confidence CREB targets. We found that this list is enriched for genes involved in cancer, and unexpectedly, highly enriched for histone genes. Furthermore, histone genes regulated by CREB were more likely to be specifically expressed in hematopoietic lineages. Decreased expression of specific histone genes was validated in K562, TF-1, and primary AML cells transduced with CREB shRNA.

**Conclusion:**

We have identified a high confidence list of CREB targets in K562 cells. These genes allow us to begin to understand the mechanisms by which CREB contributes to acute leukemia. We speculate that regulation of histone genes may play an important role by possibly altering the regulation of DNA replication during the cell cycle.

## Background

Several proto-oncogenes have been demonstrated to be deregulated in human cancer. In particular, the development of the hematologic malignancies such as leukemia, is associated with aberrant expression or function of proto-oncogenes such as c-myc, evi-1, and c-abl. Many translocations with cytogenetic abnormalities that characterize leukemias involve rearrangement of transcription factors, including AML-ETO and Nup98-hox. Some of these leukemia-associated fusion proteins predict prognosis, e.g. t(8,21), t(15,17), and inv(16) are associated with a good prognosis in acute myeloid leukemia (AML) [1]. Approximately 50% of adult patients have been noted to have specific cytogenetic abnormalities. The overall survival of patients with AML is less than 50%. Since half of the patients diagnosed with AML have normal cytogenetic profiles, it is critical to understand the molecular pathways leading to leukemogenesis.

We identified that the cyclic AMP Response Element Binding Protein (CREB) was overexpressed in the majority of bone marrow samples from patients with acute leukemia [2,3]. CREB is a leucine zipper transcription factor that is a member of the ATF/CREB family of proteins [4-6]. This transcription factor regulates proliferation, differentiation, and survival in a number of cell types, including neuronal and hematopoietic cells [4,5]. CREB has been shown to be critical in memory and hippocampal development in mice [7,8]. We previously described that CREB is phosphorylated at serine 133 downstream of signaling by the hematopoietic growth factor, Granulocyte Macrophage-Colony Stimulating Factor (GM-CSF) in myeloid cells [9-11]. We further demonstrated that CREB phosphorylation results from the activation of the Mitogen Activated Protein Kinase (MAPK) and pp90 Ribosomal S6 Kinase (pp90RSK) pathways in response to GM-CSF stimulation [9].

To understand the role of CREB in normal and neoplastichematopoiesis we investigated the expression of CREB in primary cells from patients with acute lymphoblastic (ALL) and myeloid leukemia and found that CREB was overexpressed in the majority of leukemia cells from patients with ALL and AML at the protein and mRNA levels [2,3,12]. Furthermore, overexpression of CREB was associated with a worse prognosis. We created CREB transgenic mice that overexpressed CREB in myeloid cells. These mice developed enlarged spleens, high monocyte count, and preleukemia (myeloproliferative disease) after one year. Bone marrow progenitor cells from CREB transgenic mice had increased proliferative capacity and were hypersensitive to growth factors compared to normal hematopoietic stems cells (HSCs). Overexpression of CREB in myeloid leukemia cell lines resulted in increased proliferation, survival, and numbers of cells in S phase [12]. Known target genes of CREB include the cyclins A1 and D [4,5,12,13]. Both of these genes were upregulated in CREB overexpressing cells from mice and human cell lines [4,5]. Thus, CREB is a critical regulator of leukemic proliferation and survival, at least in part, through its downstream target genes.

CREB target genes have been published on the website developed by Marc Montminy  based on ChIP chip data [14]. Additional CREB target genes were described by Impey et al. [15]. In their studies, serial analysis of chromatin occupancy (SACO) was performed by combining chromatin immunoprecipitation (ChIP) with a modification of Serial Analysis of Gene Expression (SAGE). Using a SACO library derived from rat PC12 cells, approximately 41,000 genomic signature tags (GSTs) were identified that mapped to unique genomic loci. CREB binding was confirmed for all loci supported by multiple GSTs. Of the 6302 loci identified by multiple GSTs, 40% were within 2 kb of the transcriptional start of an annotated gene, 49% were within 1 kb of a CpG island, and 72% were within 1 kb of a putative cAMP-response element (CRE). A large fraction of the SACO loci delineated bidirectional promoters and novel antisense transcripts [15]. These studies suggest that CREB binds many promoters, but only a fraction of the associated genes are activated in any specific lineage. We therefore set out to measure the functional targets of CREB in a hematopoietic model system.

Since CREB is overexpressed in bone marrow cells from patients with acute leukemia compared to normal HSCs, this provides a potential target for leukemia therapy. To this end, we stably transduced myeloid leukemia cells with CREB shRNAlentivirus[16]. CREB knockdown by 80% resulted in decreased proliferation and differentiation of both normal myeloid cells and leukemia cells *in vitro *and *in vivo *[16]. However, downregulation of CREB did not affect short-term or long-term engraftment of normal HSCs in bone marrow transplantation assays [16]. To understand the pathways downstream of CREB, we investigated genes that were differentially regulated in CREB shRNA transduced cells. In this paper, we report expression profiling of genes that were differentially regulated in CREB knockdown K562 myeloid leukemia cells and could be potential targets for development of new therapies for acute leukemia.

## Methods

### Cell lines

The following human leukemia cell lines were transduced with shRNAs: K562 (Iscoves + 10% FCS) and TF-1 (RPMI + 10%FCS + rhGM-CSF. Cells were cultured at 37°C, 5% CO2 and split every 3 to 4 days. Primary AML bone marrow samples were processed as previously described [12]. All human samples were obtained with approval from the Institutional Review Board and consents were signed, according to the Helsinki protocol.

### shRNA sequence design and constructs

The CREB specific shRNA sequences were selected and validated based on accepted parameters established by Tuschl et al. [17-19]; CREB shRNA-1, CREB shRNA-2, CREB shRNA-3. Controls included empty vector, luciferaseshRNA, and scrambled shRNA. shRNA sequences are: CREB shRNA-1(5'GCAAATGACAGTTCAAGCCC3'), shRNA-2 (5'GTACAGCTGGCTAACAATGG3'), shRNA-3 (5'GAGAGAGGTCCGTCTAATG3'), LuciferaseshRNA (5'GCCATTCTATCCTCTAGAGGA3'), Scramble shRNA (5'GGACGAACCTGCTGAGATAT3'). Short-hairpin sequences were synthesized as oligonucleotides and annealed according to standard protocol. Annealed shRNAs were then subcloned into pSICO-R shRNA vectors from the Jacks laboratory at MIT [20]. The second generation SIN vector HIV-CSCG was used to produce human shRNA vectors [21].

### Microarray analysis

Total RNA (10 μg) was extracted from K562 cells transduced with vector alone or CREB shRNA was submitted to the UCLA DNA Microarray Facility. RNA samples were labeled and hybridized by standard protocol to Affymetrix Gene Chip Human Genome U133+ Array Set HG-U133A array. Gene expression values were calculated using the MAS5 software. The expression values are quantile normalized across all arrays. We obtained the expression profiles for a control set and CREB downregulated K562 cells. A t-test is performed between the two groups to identify significantly differentially regulated genes. The analysis was performed using Matlab (Mathworks, Inc.). We find a significant number of differentially expressed genes, which are either direct or indirect targets of CREB.

To further characterize the data we have aligned CREB binding data from chromatin immunoprecipitation studies with our expression data. The chromatin immunoprecipitation data was obtained from the website [14]. To identify genes that are most significantly bound by CREB and differentially expressed in our knockdown experiment we first filtered genes by their fold change (greater than 1.5 or less than 0.7). Finally, we ranked genes according to the product of the binding and expression P value (jerry_bind_data.xls) (see Additional file 1).

We characterize these genes using three types of analyses: Ingenuity Pathway Analysis (IPA), Gene Ontology term enrichment analysis and tissue distribution. For the former analysis, we used the Ingenuity Pathways Analysis tool on the lists of significant downregulated genes. We then identified functions that were overrepresented among these genes. For the second, we used the DAVID website  to identify Gene Ontology terms that were enriched in the list.

Finally, we compute the tissue distribution of the 200 genes we identified as functional CREB targets. The tissue specific expression profiles of each gene are obtained from *HG_U133A/GNF1H and GNF1M Tissue Atlas Datasets*.[22]. We first compute the logarithm of the ratio of the expression intensity of each gene in each tissue, divided by its average intensity across all tissues. We then perform hierarchical clustering of both the genes and the tissues.

### Quantitative Real-time PCR

K562 transduced with CREBshRNA(5 × 10^6^) were lysed in Trizol and stored at -80°C prior to RNA extraction. RNA extraction was performed according to a standard protocol supplied by the manufacturer (Invitrogen) and pellets were resuspended in RNAse free water. The cDNA was transcribed with a Superscript RT III based-protocol. DNAse treatment was not performed due to the selection of intron-spanning primers. Quantitative real-time PCR was performed with the SyberGreen reagent (Bio-Rad) in triplicates and analyzed by the standard curve method standardized to the housekeeping gene beta actin[23,24].

## Results and discussion

Since CREB has pleiotropic effects on cell function and potentially activates several genes in hematopoietic and leukemia cells, we performed microarray analysis with total RNA isolated from K562 chronic myeloid leukemia cells transduced with CREB or control shRNA. The comparison of transcriptional profiles in wild type and CREB shRNA transduced K562 cells revealed a large number of differentially expressed genes (see Additional file 2). Among these genes, some are direct targets of CREB, while others are indirect targets. To infer which of these genes was potentially directly regulated by CREB, we combined the expression data with the ChIP-chip data of CREB bound promoters as demonstrated by Marc Montminy[14]. As was previously observed CREB binding sites are highly conserved across different tissues. However, these sites are activated by cAMP in a tissues specific manner. Therefore by combining these two datasets we attempted to uncover the functional CREB sites in hematopoietic tissues.

Our hypothesis for discovering functional CREB sites in hematopoietic cells is that if a gene is found to be differentially expressed in the CREB shRNA K562 transduced cells, and bound by CREB it is likely to be a direct target. To identify these genes we developed a metric that accounts for both the significance of the expression change and binding data for each gene (described in detail in Methods).

Since CREB has been described as both a transcriptional activator (when phosphorylated) and a repressor, we were interested in genes that were both up and downregulated in CREB shRNA transduced cells. The resulting rank ordered list allows us to sort genes by their likelihood of being functional CREB targets in K562 cells. It is difficult to determine, however, where to draw a threshold between the true and false targets. We have decided to restrict our analysis to the top several hundred targets that had both significant changes in expression and binding, as we deemed these to be highly enriched for true versus false targets. However, we do not claim that these are the only functional CREB targets in K562 cells, as the exact number of true targets is difficult to determine. The top down and upregulated genes revealed by this analysis are listed in Tables 1 and 2, and the full list is found in the supplementary materials.

**Table 1:** Potential CREB target genes.

Gene Name	Fold Change	CREB binding	CREB site	Gene Name	Fold Change	CREB binding	CREB site
DKFZP434G222	0.551725	3.883395	ht h	HSPC056	0.44548	1.892546	ht h
ABCG2	0.479066	2.244422	ht h	HSU79303	0.573524	1.812829	ht
ALDH2	0.5604	1.989872	none	ILVBL	0.675128	1.893295	ht h
ALDH7A1	0.62012	2.051646	h	KIAA0103	0.682528	2.620283	ht h
ALS2CR19	0.46208	1.788188	ht	HSU79303	0.573524	1.812829	ht
ANC_2H01	0.659044	1.991467	ht h	ILVBL	0.675128	1.893295	ht h
ANG	0.693535	3.287977	ht	KIAA0103	0.682528	2.620283	ht h
APLP2	0.636685	1.219917	h	KIAA0141	0.689536	3.479426	h
APPL	0.668234	1.391059	h	KIAA0408	0.595271	3.603389	none
ARFD1	0.524897	2.336962	ht	KIAA0494	0.67838	5.420821	F
BCL2L11	0.589894	3.191337	H h	KLF5	0.553523	2.062499	H
BECN1	0.600243	1.151217	H h	KNSL8	0.468603	7.854334	HT ft
BMX	0.315984	1.072006	none	KPNA5	0.562667	2.859517	none
C20orf133	0.635849	2.420642	h	LANCL1	0.647544	1.020319	none
C6orf67	0.610619	2.665053	h	LOC51668	0.500097	1.062053	ht h
CA2	0.592202	1.082939	ht	LOC51762	0.599397	3.307553	ht h
CALB2	0.671562	1.894443	h	LYPLA3	0.664078	2.379015	HT h
CCDC2	0.533032	1.529166	none	MAF	0.597194	2.383458	FT
CENPE	0.306986	3.736367	FT ht	MAPKAPK5	0.699356	2.053184	FH
CGI-77	0.664435	4.334985	H ht h	MDM2	0.468991	2.523732	none
CLDN18	0.566707	4.30699	ht h	MGC15419	0.617252	3.032433	h
CNN1	0.670957	1.150221	F ht h	MPHOSPH1	0.423771	3.535138	ht h
CREB1	0.382751	1.816762	HT H ht h	MSH2	0.592302	3.203985	h
CSPG6	0.573523	3.082765	h	MVD	0.632896	3.854905	ht h
CUL5	0.683117	2.073118	H ht h	MYL4	0.69963	1.010099	h
DBP	0.67969	2.805267	ft ht	NEFL	0.343403	2.413823	HT h
DES	0.521516	1.509794	ht h	NFKBIL1	0.695019	4.072353	ht
DIS3	0.692573	3.837304	HT ht	NIPSNAP1	0.679129	1.215594	h
DNCI1	0.673721	2.195167	none	NOX3	0.455479	2.60292	h
DNMT3A	0.679821	1.035348	h	NR4A3	0.543361	5.002146	HT H h
DSIPI	0.40458	2.546212	HT	NUDT5	0.673003	2.561752	h
DUSP19	0.674195	2.225933	none	NUMB	0.675667	1.014954	HT ht
EIF2S1	0.631867	1.075696	H ht h	PDE6B	0.66696	2.699363	h
EIF2S2	0.644661	3.313634	ht h	PEX12	0.694707	6.199684	h
ESRRBL1	0.67914	4.633352	FH h	PFDN4	0.507631	2.196535	none
FBXO22	0.688756	2.206273	ht	PHC1	0.672187	1.053985	HT
FECH	0.516446	1.045191	h	PKD2L2	0.513894	2.249593	h
FECH	0.658471	1.045191	h	PLAA	0.603854	9.235476	none
FLJ10853	0.622952	3.981514	H ht	PPP1R2	0.568734	2.04019	ft
FLJ10858	0.668758	1.523113	none	PRDX3	0.615229	1.847784	none
FLJ10904	0.54026	1.085341	none	PSAT1	0.47554	2.492965	ht
FLJ11011	0.610253	3.387879	ht h	PSMAL/GCP	0.68221	1.341117	none
FLJ11342	0.683482	2.617474	ht	PTGS2	0.684401	3.057276	ht h
FLJ11712	0.62618	2.776373	ht	RAB31	0.698664	1.12667	ht
FLJ13491	0.633125	3.268155	none	RB1CC1	0.533475	1.390318	none
FLJ20130	0.640787	2.766588	h	RFC3	0.577787	6.745001	FH ht
FLJ20331	0.681859	8.752576	H	RHEB	0.682202	3.47317	HT H h
FLJ20333	0.690542	1.946262	ht h	RNASE4	0.436168	2.975774	ht h
FLJ20509	0.691949	1.96435	none	SARS2	0.692149	5.455469	H h
FLJ23233	0.471676	1.517415	none	SBBI26	0.683312	6.75719	H
FOXD1	0.593522	5.160553	HT ht	SDP35	0.502432	2.320591	h
GCAT	0.656744	2.122675	ht h	SERPINI1	0.31594	3.277692	ht
GCHFR	0.676365	2.188753	ht h	SHMT1	0.658252	1.127084	ht h
GFI1B	0.671179	0.999255	h	SILV	0.662805	2.130617	H
GMPR	0.672975	1.149663	ht	SLC11A2	0.684325	1.842417	none
GOLGA4	0.567882	2.939327	ht h	SLC22A5	0.657746	1.64513	none
GPNMB	0.410992	1.004344	none	SLC27A6	0.547039	1.029816	ht
GRHPR	0.68706	2.454475	H ht	SLC2A4	0.507466	2.273185	ht h
H2BFS	0.591569	2.358423	ht	SLC39A8	0.201136	1.004832	none
HBE1	0.639376	0.947159	h	SLC4A7	0.532067	1.262531	ht
HDGFRP3	0.65013	1.208322	none	SMARCA1	0.519982	1.056916	HT ht
HDGFRP3	0.668211	1.208322	none	SMC2L1	0.596288	2.916083	ht h
HEXA	0.54467	2.622927	none	SRI	0.671893	0.826457	ht
HIST1H1C	0.590374	1.983514	h	STK16	0.680797	6.555535	H h
HIST1H2AD	0.66909	4.768013	ht h	SULT1C2	0.599235	3.511947	f h
HIST1H2AI	0.542518	2.801688	H ht h	SURB7	0.498245	1.598812	ht
HIST1H2AJ	0.696531	3.066865	ft ht h	SYN1	0.696375	3.016534	F h
HIST1H2AL	0.602018	2.600144	FHT ht h	TAF1A	0.589389	2.689618	none
HIST1H2BB	0.590821	1.782458	ht h	TBC1D7	0.692755	1.281463	ht
HIST1H2BD	0.674855	3.111055	HT ht h	TCTE1L	0.368312	2.475611	ht
HIST1H2BE	0.546621	2.34815	ht	TFDP2	0.670657	1.016413	ht
HIST1H2BF	0.543665	1.985466	ht	TGDS	0.67197	1.523411	none
HIST1H2BH	0.617917	2.04185	none	THRB	0.670555	2.256453	H ht h
HIST1H2BI	0.585897	1.443622	ht	TMEM14A	0.656093	1.175355	ht h
HIST1H2BJ	0.493823	5.335159	HT ht h	TOM1	0.64031	3.221137	h
HIST1H2BM	0.687469	3.533372	ft ht h	TXN2	0.689274	1.893339	H ht h
HIST1H2BO	0.618862	4.014214	ht h	UBE2B	0.663194	3.652863	H ht h
HIST1H3B	0.556438	4.260113	ft ht	VRK1	0.650583	1.000406	h
HIST1H3H	0.641946	2.647758	H ht h	WASPIP	0.572355	1.01892	none
HIST1H4E	0.608257	2.458831	FT h	WDHD1	0.624889	4.984045	H ht h
HIST1H4I	0.612088	2.068983	ht	WWOX	0.671866	1.882778	h
HIST2H2AA	0.560962	4.032876	ht	ZNF134	0.677481	2.726853	ht h
HLA-DRA	0.365141	3.086303	ht h	ZNF222	0.5618	4.09755	ht h
HLXB9	0.667926	1.006593	none	ZNF230	0.410725	3.76825	ht h
HS2ST1	0.694429	1.032562	ht h	ZNF235	0.38371	2.959812	none
HSBP1	0.671929	1.891961	ht h				

Top down-regulated genes that show significant CREB binding and changes in expression in the CREB knockdown cells. The detailed criteria for selecting these genes are described in the methods section. For each grouping of genes, from left to right, column 1 shows the gene symbols, column 2 the ratio of the expression change in wild type versus knockdown, column 3 the CREB binding ratio and column 4 the presence of CREB binding motifs. The key for column 4 is as follows: F is a full CREB motif (TGACGCTA) that is conserved from human to mouse, while f is not conserved, H is a conserved CREB half motif (TGACG or CGTCA), while h is not conserved, and T is the conserved presence of a TATA motif less than 300 base pairs downstream of the CREB motif, while t is not conserved.

**Table 2:** Potential CREB target genes.

Gene Name	Fold Change	CREB binding	CREB site	Gene Name	Fold Change	CREB binding	CREB site
ACOX1	2.110674	2.911283	H ht	LDLR	1.678587	1.525499	ht
ADAT1	1.410234	3.769574	ht f h	LGALS3BP	2.131291	3.615437	none
APEH	1.400261	2.527266	h	LIM	1.696177	1.097432	none
APPBP2	1.486616	2.151867	H ht h	LIM	1.849989	1.097432	none
ARHB	2.758453	2.77377	H ht	LRRFIP1	1.941595	1.122307	h
ATP6V1A	1.446867	3.016595	HT ht h	METAP2	1.916632	2.635425	ht
BCL6	1.640646	6.084626	HT ht	METTL2	1.593867	3.474639	none
BDKRB2	1.600927	2.601219	none	MGC2731	1.588545	2.80081	HT h
BTN3A2	1.465264	3.426679	ht	MGC4054	1.502743	2.777966	ht
C20orf12	1.511854	3.12999	h	MOCS3	1.796255	5.213295	none
C20orf121	1.456022	3.532969	H	MRPS10	1.410471	1.834794	ht f
C20orf172	1.463616	4.659037	H h	NCOA3	1.495237	2.715807	ht
C20orf23	1.528396	2.622103	none	NDRG1	2.030896	2.312257	ht h
CD44	9.531947	1.335178	ht h	NEDF	1.567662	4.268912	ft ht
CDH12	3.296441	1.178959	none	NPR2L	1.618864	6.397355	ht h
CDKAL1	1.735322	3.445022	none	ODZ1	1.448279	2.310975	ht
CDKN1A	2.216725	1.778747	H ht h	OPA3	1.474233	7.631458	FHT ht h
CELSR3	1.546375	3.175919	H ht	OTC	1.693003	4.881484	ht
CENPF	1.415064	2.654622	ht	PAFAH2	1.67217	4.584628	none
CHRNB1	1.55045	1.412576	H h	PAFAH2	1.631066	4.584628	none
CLECSF2	1.747573	1.251667	none	PHC3	1.42261	1.747154	ht
CML2	1.47905	3.427882	ht	PHLDA1	3.92008	2.003171	h
COL15A1	2.56792	1.394566	none	PLAT	1.668223	1.95203	none
CREM	1.793497	3.67068	H	PLEKHB2	1.568395	4.611748	f
CRKL	1.690269	3.051845	H h	PPARGC1	2.268458	2.972107	HT F ht h
CSMD1	1.647116	1.61907	ht	PPFIBP1	1.852526	2.550633	ht h
CTMP	1.548763	3.386235	none	PPP1R10	1.870902	2.447557	H h
DBT	1.518604	4.292329	none	PPP1R3B	1.693114	1.622596	h
DCLRE1C	1.41992	3.010944	none	PSMAL/GCP	1.506527	2.707076	none
DDOST	1.582101	2.508459	ht	RAB7L1	1.638378	1.15364	ht h
DDX3X	1.817009	3.42975	none	RABL2B	1.486054	2.496157	h
DEGS	1.488221	1.464348	none	RASSF1	1.431271	4.04395	none
DIAPH1	1.412484	2.96506	none	RBL1	1.529652	2.451247	h
DUSP1	1.578824	2.102797	FT HT ht h	REL	1.944847	1.143935	H h
EGR2	5.148023	2.036633	HT ht h	RHOBTB3	1.63057	2.813465	none
EIF5	1.422558	4.208549	ht h	RIOK3	1.40951	2.008376	none
ELK1	1.405171	4.088789	ht	RNASE6PL	1.561704	2.252099	ht
ENC1	1.957151	1.549567	h	RNF32	1.954396	1.603905	H ht
F2R	1.804785	1.098488	ht h	SAS	1.768493	7.735178	HT ht h
FAM13A1	1.780869	2.014276	none	SERPINB9	2.244605	1.418097	ht h
FAT	2.00051	1.816506	F ht	SFPQ	1.477265	3.428149	ht
FKBP14	1.78994	3.042488	ht	SHARP	1.558516	1.078188	H ht
FLJ10781	1.463332	1.113364	ht h	SLC31A1	1.491104	3.803168	FH ht
FLJ10803	1.726196	2.63943	ht	SLC35E3	1.716026	1.969928	ht
FLJ11029	1.422001	3.085667	ht h	SLC38A2	1.497716	1.914154	H ht
FLJ11151	2.413055	1.840398	h	SLC39A6	1.477678	3.119807	h
FLJ20507	1.730068	2.922871	H ht h	SMA3	1.414595	2.654203	ht
FOSL1	2.220086	1.929543	HT ht h	SMARCF1	1.537978	1.046929	none
FRSB	1.423607	2.982919	ht	SNAP29	1.521481	2.454502	h
FXC1	1.423019	5.02095	HT H ht	SON	1.42477	4.933417	H
GALNS	1.772331	2.592543	h	SPG4	1.413533	3.160161	none
GCA	1.690161	2.92801	H h	SUFU	1.661693	2.275704	ht h
GTF2H3	1.593421	10.587057	H	TAP1	1.435113	3.105625	H h
GYS1	1.418699	2.559154	h	TIGD6	1.772719	3.636168	h
HBS1L	1.475369	3.891767	ht	TIMP1	1.791155	1.848154	HT h
HIP1	1.537214	2.114631	ht h	TNFRSF21	1.498482	2.635088	ht
HLA-C	1.429002	3.2916	h	TP53AP1	1.527339	3.493111	ht h
HSPG2	1.708361	1.453039	none	TPM4	2.201468	1.33368	H ht
ICAM1	2.20462	1.198603	ht h	TRIM26	1.400065	6.12308	ht
ID1	1.521685	2.3068	FT ht	TSSC3	1.879281	2.01021	H ht h
IDS	1.508286	1.1848	h	TTF1	1.513382	3.461645	ht h
IER5	1.66867	2.847755	HT ht	TUBA3	1.481437	2.500545	none
IL10RA	1.64246	2.830231	f	U2AF1L1	2.758542	3.548509	ht
IL10RB	1.410005	1.192048	ht h	U5-116KD	2.223148	2.779884	h
IL1R1	1.812093	1.329947	ht	USP2	2.35423	3.920336	HT H h
IL6	1.980266	1.460112	HT ht	VPS4B	1.474465	6.693871	H ht
IL6ST	1.54702	3.418269	none	YME1L1	1.441837	1.843132	F ht h
INPP1	2.071508	1.550135	ht h	ZFP37	1.572207	4.659572	ht h
ITGA5	2.028008	1.315131	none	ZNF142	1.50914	3.028386	h
JM4	1.606813	2.392743	HT h	ZNF155	1.69746	4.195939	none
KIAA0266	1.504796	2.986155	none	ZNF189	1.625836	4.104303	ht h
KIF14	1.453888	4.181899	none	ZNF221	1.777122	3.569536	none
KIF3B	1.623133	1.560467	none	ZNF324	1.488601	4.205703	h
LCMT2	1.587221	2.338943	H ht h				

Top up-regulated genes that show significant CREB binding and changes in expression in the CREB knockdown cells. The detailed criteria for selecting these genes are described in the methods section. The column descriptions are the same as in Table 1.

**Figure 1:**
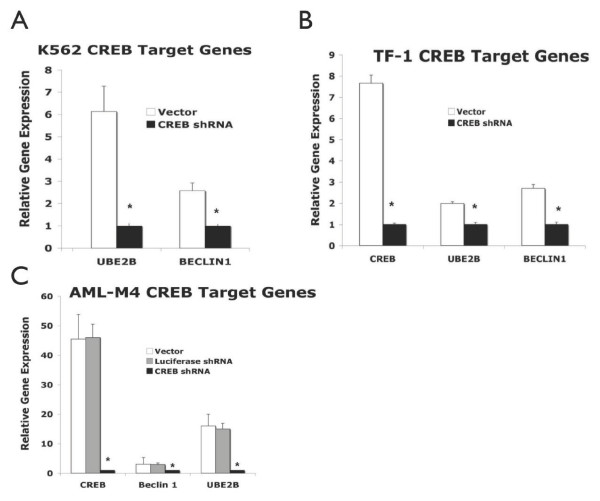
**Expression of potential target genes downstream of CREB in myeloid leukemia cells. Primers specific for the UBE2B, BECLIN1, and CREB genes were generated and utilized for quantitative real-time PCR by SyberGreen method (Bio-Rad Inc.)** Relative gene expression normalized to the housekeeping gene actin is shown for the following transduced cells: (A) K562 myeloid leukemia cells, (B) TF-1 myeloid leukemia cells, and (C) Human AML-M4 blasts.

Genes within the downregulated list were BECLIN 1, UBE2B. Both these genes have a cAMP responsive element binding site(s) in their promoters. These genes were selected for further validation because they are known to be involved in autophagy/apoptosis (BECLIN 1), cell cycle/DNA repair (UBE2B) [25-28]. Quantitative real time-polymerase chain reaction (qRT-PCR) with mRNA from AML cell lines (K562 and TF-1) and primary leukemic blasts from a patient with M4-AML was performed. UBE2B expression was significantly reduced in CREB shRNA transduced TF-1 and K562 myeloid leukemia cells compared to controls (Figure 1, p < 0.05). BECLIN and UBE2B were downregulated in primary AML cells transduced with CREB shRNA (Figure 1, p < 0.05).

Having confirmed the validity of our microarray results in these two test cases we set out to characterize the function of the complete list of CREB target genes using two annotation schemes. The first utilizes the annotation contained in the Ingenuity Pathway Analysis software (IPA). This analysis showed that there is a significant enrichment for cell cycle (P < 1e-3) and cancer (P < 1e-3) genes. The full list of genes associated with cancer is shown in Table 3. Many of these genes regulate cell cycle, signaling, DNA repair, or metabolism, which are consistent with previously published results [5,15]. Furthermore, the role of CREB in the pathogenesis of leukemias has also been described in the literature [2,3,12,29].

**Table 3:** The subset of CREB target genes associated with cancer according to Ingenuity Pathways Analysis.

Name	Location	Type	Drugs
Downregulated Cancer Genes			
ABCG2	Plasma Membrane	transporter	
ANG	Extracellular Space	enzyme	
BCL2L11	Cytoplasm	other	
BECN1	Cytoplasm	other	
BMX	Cytoplasm	kinase	
CA2	Cytoplasm	enzyme	methazolamide, hydrochlorothiazide, acetazolamide, trichloromethiazide, dorzolamide, chlorothiazide, dorzolamide/timolol, brinzolamide, chlorthalidone, benzthiazide, sulfacetamide, topiramate
CENPE	Nucleus	other	
CNN1	Cytoplasm	other	
CREB1	Nucleus	transcription regulator	
CUL5	Nucleus	ion channel	
GFI1B	Nucleus	transcription regulator	
KLF5	Nucleus	transcription regulator	
MDM2 (includes EG:4193)	Nucleus	transcription regulator	
MPHOSPH1	Nucleus	enzyme	
MSH2	Nucleus	enzyme	
MVD	Cytoplasm	enzyme	
NR4A3	Nucleus	ligand-dependent nuclear receptor	
NUMB	Plasma Membrane	other	
PPP1R2	Cytoplasm	phosphatase	
PTGS2	Cytoplasm	enzyme	acetaminophen/pentazocine, acetaminophen/clemastine/pseudoephedrine, aspirin/butalbital/caffeine,
RB1CC1	Nucleus	other	
SILV	Plasma Membrane	enzyme	
SMC2	Nucleus	transporter	
SMC3	Nucleus	other	
TFDP2	Nucleus	transcription regulator	
THRB	Nucleus	ligand-dependent nuclear receptor	3,5-diiodothyropropionic acid, amiodarone, thyroxine, L-triiodothyronine
UBE2B	Cytoplasm	enzyme	
VRK1	Nucleus	kinase	
WWOX	Cytoplasm	enzyme	
Upregulated cancer Genes			
ACOX1	Cytoplasm	enzyme	
ARID1A	Nucleus	transcription regulator	
BCL6	Nucleus	transcription regulator	
BDKRB2	Plasma Membrane	G-protein coupled receptor	anatibant, icatibant
CD44	Plasma Membrane	other	
CDKN1A	Nucleus	kinase	
COL15A1	Extracellular Space	other	collagenase
CREM	Nucleus	transcription regulator	
CRKL	Cytoplasm	kinase	
DCLRE1C	Nucleus	enzyme	
DEGS1	Plasma Membrane	enzyme	
DIAPH1	Cytoplasm	other	
DUSP1	Nucleus	phosphatase	
EGR2	Nucleus	transcription regulator	
ELK1	Nucleus	transcription regulator	
ENC1	Nucleus	peptidase	
F2R	Plasma Membrane	G-protein coupled receptor	chrysalin, argatroban, bivalirudin
FOSL1	Nucleus	transcription regulator	
HIP1	Cytoplasm	other	
HSPG2 (includes EG:3339)	Plasma Membrane	other	
ICAM1	Plasma Membrane	transmembrane receptor	
ID1	Nucleus	transcription regulator	
IL6	Extracellular Space	cytokine	tocilizumab
IL1R1	Plasma Membrane	transmembrane receptor	anakinra
IL6ST	Plasma Membrane	transmembrane receptor	
ITGA5	Plasma Membrane	other	
KIF14	Cytoplasm	other	
METAP2	Cytoplasm	peptidase	PPI-2458
NCOA3	Nucleus	transcription regulator	
NDRG1	Nucleus	kinase	
PHLDA1	Cytoplasm	other	
PLAT	Extracellular Space	peptidase	
RASSF1	Nucleus	other	
RBL1	Nucleus	other	
REL	Nucleus	transcription regulator	
RHOB	Cytoplasm	enzyme	
SERPINB9	Cytoplasm	other	
SUFU	Nucleus	transcription regulator	
TIMP1	Extracellular Space	other	
TNFRSF21	Plasma Membrane	other	
USP2	Cytoplasm	peptidase	

Column 1 is the gene name, column 2 the localization, column 3 is a description of the protein function and column 4 are compounds that target the protein.

**Figure 2:**
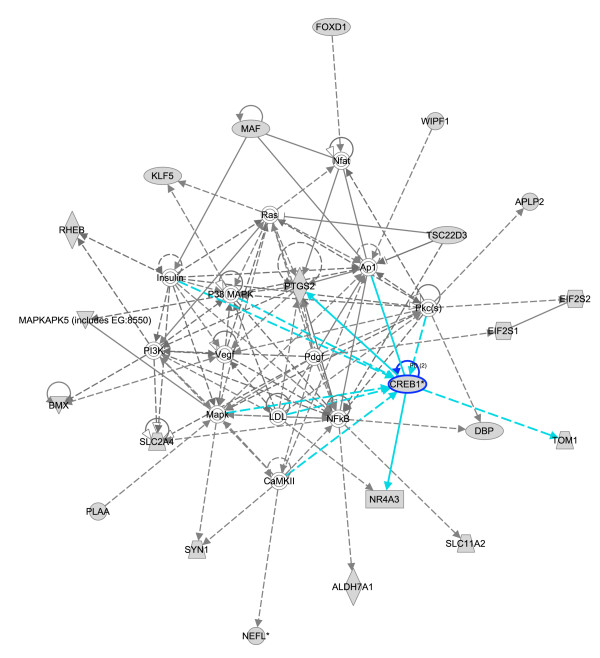
**A network depicting interactions between direct CREB targets (shown in grey) and proteins that these interact with (shown in white).** PTGS2, NR4A3 and TOM1 are direct CREB targets whose regulation by CREB was previously described in the literature (clue lines). PTGS2 (COX2) emerges as a central player in this network, and is thus implicated as a potential regulator of leukemias.

IPA also allows us to study CREB target genes in the context of protein-protein interactions networks. A network for downregulated genes interacting with CREB is shown in Figure 2, with a subset of the downregulated targets shown in grey, while other genes not in the target list that interact with these, shown in white. Here we see that there is prior literature supporting our analysis that CREB1 regulates PTGS2 (COX2), NR4A3 and TOM1, as depicted by the blue lines. Interestingly, COX2 is an important drug target, and suggests that commonly used COX2 inhibitors may provide a target for acute leukemia.

The second analysis that we performed used the terms from Gene Ontology to identify common characteristics among the top K562 CREB targets. Here we find the striking and unexpected result that ten percent of the downregulated targets code for histone genes (P < 1e-10, Table 4). We also performed an analysis of the top upregulated genes but did not find any significant GO terms. Although there is some prior literature indicating that CREB or CREB-related pathways may play a role in regulating histone modifications primarily through the histone acetylase CREB Binding Protein (CBP)[5,30,31], the fact that CREB directly regulates the transcription of histone genes in these cells is unexpected.

**Table 4:** Gene Ontology terms that are enriched among the top CREB targets.

Category	Term	Count	%	PValue
GOTERM_CC_ALL	nucleosome	11	6.88%	6.22E-10
GOTERM_CC_ALL	chromosome	17	10.62%	2.39E-09
GOTERM_BP_ALL	nucleosome assembly	11	6.88%	6.60E-09
GOTERM_CC_ALL	chromatin	13	8.12%	7.56E-09
GOTERM_BP_ALL	chromatin assembly	11	6.88%	1.66E-08
GOTERM_BP_ALL	protein complex assembly	15	9.38%	2.19E-07
GOTERM_BP_ALL	chromatin assembly or disassembly	11	6.88%	3.84E-07
GOTERM_BP_ALL	chromosome organization and biogenesis	15	9.38%	5.56E-07
GOTERM_BP_ALL	chromosome organization and biogenesis (sensu Eukaryota)	14	8.75%	1.63E-06
GOTERM_CC_ALL	membrane-bound organelle	75	46.88%	1.93E-06
GOTERM_CC_ALL	intracellular membrane-bound organelle	74	46.25%	4.63E-06
GOTERM_CC_ALL	organelle	83	51.88%	5.39E-06
GOTERM_MF_ALL	DNA binding	38	23.75%	6.17E-06
GOTERM_BP_ALL	cellular physiological process	118	73.75%	8.86E-06
GOTERM_BP_ALL	establishment and/or maintenance of chromatin architecture	12	7.50%	1.02E-05
GOTERM_CC_ALL	intracellular organelle	82	51.25%	1.28E-05
GOTERM_BP_ALL	DNA packaging	12	7.50%	1.38E-05
GOTERM_BP_ALL	organelle organization and biogenesis	22	13.75%	1.59E-05
GOTERM_CC_ALL	nucleus	56	35.00%	2.46E-05
GOTERM_BP_ALL	DNA metabolism	19	11.88%	2.63E-05

Column 1 is the ontology used (BP is biological process, CC is cellular localization and MF is molecular function), column 2 is the term, column 3 is the number of genes in the target list associated wit the term, column 4 is the percentage of genes in the target list associated with the term and column 5 is the P value for observing this number genes associated with the term.

To further validate the hypothesis that CREB is an activator of these 20 histone genes, we utilized previously published analyses of the gene promoters to identify consensus CREB binding sequences. The results shown in Table 1 demonstrate that nearly all the histone genes contain CREB half sites along with a TATA box in the vicinity of these. Thus three lines of evidence support the assignment of these 20 histone genes as CREB targets in K562 cells: expression, binding and sequence based.

**Figure 3:**
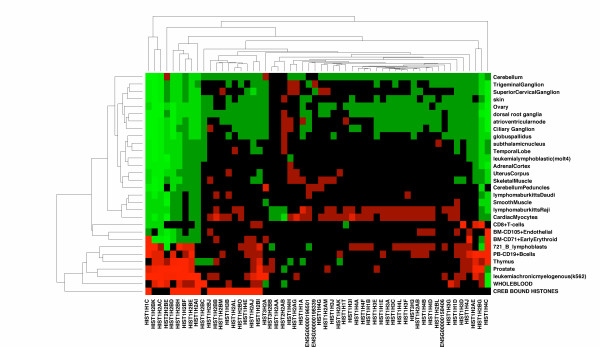
**The tissue specific expression of histone genes. Each row of the figure represents a tissue from the GNF Body Atlas (see methods). **We show only the top 30 tissues with highest variance of expression of histone genes. Each column represents a histone gene. We use hierarchical clustering to order the rows and columns according to their similarity. Red indicates that the gene is over expressed relative to its mean expression levels across all tissues, and green that it is under expressed. The histone genes that we identify as direct targets of CREB are shown in red in the last row of the figure. We see that many of these are only expressed in a small subset of rapidly dividing tissues along with K562 cells.

We examined the distribution of expression of these 20 histone genes across human tissues. The expression data were obtained from the GNF body atlas. We were able to extract expression profiles for 81 histone genes contained in the human genome. Fifteen of these overlapped with the 20 histone CREB targets. We show the expression of all 81 histone genes in Figure 3, where the identity of the 15 CREB target genes is shown in the last row. We see that the 15 genes are clustered into two groups containing more than one gene, with a third group consisting of a single histone HIST1H1C. One of the groups contains histones that are broadly expressed across human tissues, and particularly in all hematopoietic tissues. The second group is instead expressed in a very narrow range of tissues including K562 cells, bone marrow, prostate and thymus.

**Figure 4:**
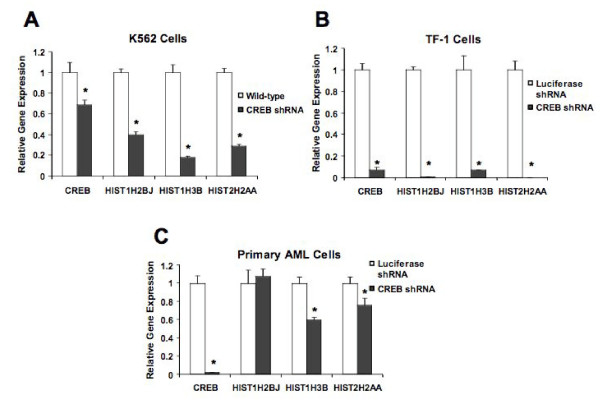
**Expression of target histone genes is decreased in CREB knockdown myeloid leukemia cells. Primers specific for HIST1H2BJ, HIST1H3B, and HIST2H2AA were generated and utilized for quantitative real-time PCR by the SYBR Green method (Applied Biosystems).** Relative gene expression normalized to the housekeeping gene actin is shown for the following transduced cells: (A) K562 myeloid leukemia cells, (B) TF-1 myeloid leukemia cells, and (C) primary AML cells.

We examined the expression of three histones that are putative targets of CREB by real time PCR with mRNA from K562, TF-1, and primary cells from patients with AML. The three histones selected were based on our microarray analyses. Our results demonstrated a statistically significant decrease in histonesHIST1H2Bj, HIST1H3B, and HIST2H2AA in K562 and TF-1 cells (Figure 4). Interestingly, in primary cells from a patient with AML, only HIST1H3B and HIST2H2AA, but not HIST1H2BJ expression was decreased with CREB knockdown. These results suggest that histones are differentially expressed in AML and that specific histones are potential targets of CREB. This analysis supports the hypothesis that CREB regulates a subset of histone genes that are normally expressed in a small set of rapidly dividing tissues. These genes are presumably aberrantly activated in K562 and other leukemia cells, and could potentially contribute to the malignant phenotype.

## Conclusion

We have identified a high confidence list of CREB target genes in K562 myeloid leukemia cells. Several important CREB target genes that function in DNA repair, signaling, oncogenesis, and autophagy were identified. These genes provide potential mechanisms by which CREB contributes to the pathogenesis of acute leukemia. Expression of the genes beclin-1 and ube2b was found to be decreased in myeloid leukemia cell lines and primary AML cells in which CREB was downregulated. In addition, we speculate that CREB may have more global effects on transcription, primarily through the regulation of histone genes thereby altering the regulation of DNA replication during the cell cycle.

## Competing interests

The authors declare that they have no competing interests.

## Authors' contributions

MP and SFN analyzed the microarray data, performed the statistical analysis, and drafted the manuscript. JCC, JC, DJ, and JT performed the real-time PCR experiments. KMS supervised the experiments and wrote the manuscript. All authors read and approved the final manuscript.

## Pre-publication history

The pre-publication history for this paper can be accessed here:



## Supplementary Material

Additional File 1 Supplementary table 1 Click here for file

Additional File 2 Supplementary table 2 Click here for file
